# Hospital Expenditure

**Published:** 1902-09-13

**Authors:** 


### EDITOR'S LETTER-BOX.

[Our Correspondents are reminded that prolixity is a great bar to publication, and that brevity of style and conciseness of statement greatly facilitate early insertion.]

### "HOSPITAL EXPENDITURE."

A "PUZZLED MATRON" would be glad to know the quantities allowed, the cost of milk per gallon, and what the beef tea is made of to bring "low diet" down to 4d. a day per head.

[The estimate of 4d. a day for low diet is for patients exclusively on milk who would receive 3 pints a day. The cost, reckoning the milk at 10d. a gallon (which is higher than what is given in many hospitals) would be 3¾d. and ¼d. might be added for bread or biscuit.

On page 61 of "The Hospital Commissariat" the quantities usually given for low diet are accurately worked out. Beef tea at 4d. per pint (1 lb. of meat to 1 pint), broth at 2d. a pint, milk at 10d. a gallon, bread 1d. a lb.

Example: Low diet London Hospital—

Bread, 10 ozs., ⅝d.<br>

Milk, 2 pints, 2½d. } cost, 5¼d.<br>

Broth, 1 pint, 2d.

If pudding (at ⅞d.) were substituted for broth as in some hospitals the cost would be 4d. It is only when the item of beef tea is omitted that the cost can be reduced as low as 4d. The average cost of low diet is from 4d. to 6d. per head per day. At the Birmingham General Hospital analysis of the diets showed that out of 214 occupied beds an average of 27 patients in one quarter were on milk diet (3 pints a day), an average of 57 on low diet (bread, milk and pudding), and only 3 on "middle diet" which there includes broth and beef tea.

I hope these figures are sufficient for the "Puzzled Matron." She will, of course, understand that the term "low diet" stands for almost every variety of menu excluding only meat, fish or poultry.—The AUTHOR of "Hospital Expenditure."]

